# The Use of Olkuska Sheep Milk for the Production of Symbiotic Dairy Ice Cream

**DOI:** 10.3390/ani12010070

**Published:** 2021-12-29

**Authors:** Magdalena Kowalczyk, Agata Znamirowska, Małgorzata Pawlos, Magdalena Buniowska

**Affiliations:** Department of Dairy Technology, Institute of Food Technology and Nutrition, University of Rzeszow, Cwiklinskiej 2D, 35-601 Rzeszow, Poland; aznamirowska@ur.edu.pl (A.Z.); mpawlos@ur.edu.pl (M.P.); mbuniowska@ur.edu.pl (M.B.)

**Keywords:** sheep milk, Olkuska breed, dairy ice cream, probiotics, symbiotic, apple fiber, *Lactobacillus*, *Lacticaseibacillus*

## Abstract

**Simple Summary:**

Ice cream may be used as a carrier to deliver probiotics and prebiotics. In this study, we decided to investigate the possibility of using sheep milk from the Olkuska breed for ice cream manufacture and evaluate the viability of *Lactobacillus* and *Lacticaseibacillus* strains and the chemical, physical and organoleptic properties of dairy ice cream during storage. The obtained results contribute to a more practical application of different probiotic strains for the fermentation of ice cream mixes and the possibility of using apple fiber in ice cream production. Moreover, the study’s findings showed that symbiotic ice cream with acceptable physicochemical and organoleptic characteristics might be produced from sheep milk of the Olkuska breed.

**Abstract:**

The aim of this study was to determine the possibility of using Olkuska sheep milk for the production of ice cream with probiotics and prebiotics. The study examined the effect of the storage and type of bacteria used for the fermentation of ice cream mixes and partial replacement of inulin with apple fiber on the physicochemical properties, viability of probiotic cultures and organoleptic properties of sheep’s milk ice cream stored at −22 °C for 21 days. The addition of apple fiber reduced the pH value of ice cream mixes before fermentation. In ice cream mixes and ice cream with apple fiber, the lactic acid content was higher by 0.1–0.2 g L^−1^ than in their equivalents with inulin only. These differences persisted during the storage of the ice cream. After fermentation of the ice mixes, the bacterial cell count ranged from 10.62 log cfu g^−1^ to 12.25 log cfu g^−1^. The freezing process reduced the population of probiotic bacteria cells in ice cream with inulin from 0.8 log cfu g^−1^ in ice cream with *Lactobacillus acidophilus*, 1.0 log cfu g^−1^ in ice cream with *Lacticaseibacillus paracasei* and 1.1 log cfu g^−1^ in ice cream with *Lacticaseibacillus* *casei*. Freezing the varieties with apple fiber also resulted in a reduction of viable bacterial cells from 0.8 log cfu g^−1^ in ice cream with *L. paracasei* and *Lb. acidophilus* to 1 log cfu g^−1^ in ice cream with *L. casei*, compared to the results after fermentation. The highest percentage overrun was determined in ice cream with *L. paracasei* and *Lb. acidophilus*. Ice cream with *L. casei* was characterized by significantly lower overrun on the 7th and 21st days of storage. Although *L. paracasei* ice cream had the highest overrun, it did not cause a significant reduction in the probiotic population during storage. After seven days of storage, the first drop differed significantly depending on the type of bacteria used for fermentation of the mixture and the addition of apple fiber. *L. casei* ice cream had a longer first drop time than *L. paracasei* and *Lb. acidophilus* ice cream. Partial replacement of inulin with apple fiber resulted in a significant darkening of the color of ice cream mixes. Depending on the type of bacteria used for fermentation, the addition of apple fiber decreased the value of the L* parameter. Ice cream mixes and ice cream with inulin and apple fiber were characterized by a high proportion of yellow. Partial replacement of inulin with apple fiber reduced the hardness of ice cream compared to inulin-only ice cream. Moreover, the panelists found that ice cream with inulin was characterized by a sweeter taste than ice cream with apple fiber. Moreover, the addition of apple fiber favorably increased the flavor and aroma perception of the mango-passion fruit. Therefore, the milk of Olkuska sheep could be successfully used for the production of symbiotic dairy ice cream.

## 1. Introduction

The parameters of sheep milk are influenced by various factors including genetic, physiological and environmental. Polish sheep breeds, such as Olkuska, are well adapted to local environmental conditions. This breed is characterized by resistance to diseases and demanding environmental conditions, moreover good milk yield [1].

Sheep milk is characterized by a higher content of total solids compared to cow and goat milk. Moreover, sheep milk is characterized by a high content of micro- and macroelements, vitamins, protein and fat [2].

The protein content of raw milk differs among the species and the sheep raw milk has the higher protein content (5.5%). According to Balthazar et al. [3] the total casein content in sheep raw milk is 85% of total proteins, where αS_1_, αS_2_, β and κ-casein represent 6.7%, 22.8%, 61.6% and 8.9%, respectively. The remaining 15% of milk proteins includes major whey proteins β-lactoglobulin and α-lactalbumin as well as other protein constituents [4].

Milk proteins have a wide range of functional properties such as emulsifying, thickening, gelling and foaming. Milk proteins promote the formation and stabilization of oil droplets in emulsions or air bubbles in foams in food formulations [5]. Moreover, the higher viscosity of sheep milk may also be attributable to an increased water binding capacity in the milk proteins [6]. These functional properties of milk proteins are used to produce dairy products such as ice cream.

Raw milk from different species remains an element of human nutrition. Therefore, the free and total amino acid profile of milk from different species plays a crucial role for both milk producers and processors as well as consumers to achieve innovative new product design, complexity, flavor and functionality [4]. The characteristic profiles of free amino acid content vary among species. According to Landi et al. [4], the free amino acid found in the highest amounts in raw cow, sheep and goat milk is glutamic acid (9.07 mg per 100 g), tyrosine (4.72 mg per 100 g) and glycine (4.54 mg per 100 g). In contrast, raw goat milk is a rich source of taurine (14.92 mg per 100 g), which is found in small amounts in raw cow milk (1.38 mg per 100 g) and sheep milk (2.10 mg per 100 g).

Sheep milk could be considered a substitute for cow’s milk for allergy sufferers. Specific antibodies in milk-allergic individuals (IgE) poorly recognize the αS1-casein, αS2-casein and β-casein protein fractions from goat and sheep milk, which is not observed with cow milk [3,7]. Nevertheless, protein polymorphism is described as playing an essential role in the induction of different degrees of an allergic reaction [3,8].

Sheep’s milk ice cream can be an excellent carrier of bioactive compounds, including probiotic cultures and prebiotic ingredients. The high protein level and fat in sheep’s milk allows for the production of ice cream with a higher density, which translates into better protection of probiotic cells against oxygen access during ice cream storage and during passage through the gastrointestinal tract [9].

Probiotics added to food benefits by restoring the intestinal microflora balance [10,11,12] with a minimum number of live probiotic cells of 10^6^–10^9^ cfu g^−1^ is required for a therapeutic effect [13,14,15]. Probiotic bacteria used in ice cream production should be freeze-resistant and survive for a particular time at low temperatures [16,17,18]. *Lactobacillus* and *Bifidobacterium* are the most commonly used probiotic strains in food production [19,20]. Few studies show that probiotics in milk ice cream have better survival in the presence of inulin [21,22]. Inulin can be used as a replacement for fat and sugar, improving the viscosity of ice cream, thus affecting better overrun, resistance to melting and maintaining shape by stabilizing the foam [23] due to the ability to bind water and create a gel network, giving a feeling of smoothness and creaminess [24,25] in addition, it reduces the hardness of reduced-fat ice cream [26].

Recent studies indicate that prebiotic compounds may also increase the bioavailability of milk protein [27]. Simultaneous consumption of prebiotic ingredients that ferment into acetate, propionate, and butyrate stimulates the growth and development of probiotic bacteria [28]. Fruit fibers are gaining more and more recognition among consumers and are considered natural additives with a low degree of processing [29]. This group of prebiotics includes apple fiber, a natural fruit product containing protein, carbohydrates, polyphenols and pectins [30]. The addition of fiber to dairy products fits well with the “clean label” trend in the natural food market segment.

Sheep milk ice cream with the addition of prebiotics such as inulin and apple fiber, apart from the health benefits and properties stimulating the growth of probiotic bacteria, is an innovative product with functional properties. There are few publications on the production of ice cream from sheep’s milk with probiotic strains and prebiotics in the scientific literature. Therefore, the study aimed to determine the possibility of using Olkuska sheep milk for the production of ice cream with probiotics and prebiotics.

## 2. Materials and Methods

### 2.1. Materials

Raw morning and cooled (4 °C) sheep’s milk for the production of ice cream was purchased from the farm “Owcza Zagroda” (Wyżne, Podkarpacie, Poland) in June 2021. A flock of 35 Olkuska breed sheep were kept on the farm. Green fodder and hay with the addition of cereals (oats, barley and maize in a quantity of 0.5 kg/sheep/day) from the breeder’s farm were used for feeding animals. Milking was conducted by hand. Before ice cream manufacture, milk was filtrated to remove dirt and foreign particles.

The chemical composition of milk was determined in the chemical composition analyzer of milk and dairy products Bentley B-150 (Bentley, MN, USA).

The following materials were used to prepare the ice cream mixture: inulin (carbohydrate 97 g/100 g, including sugars 7 g/100 g, fiber 90 g/100 g, fat 0 g/100 g and protein 0 g/100 g; Orafti HP, Oreye, Belgium); apple fiber (carbohydrate 87 g/100 g, including sugars 27 g/100 g, fiber 51 g/100 g, fat 3.3 g/100 g and protein 5.1 g/100 g; Aura Herbals Jarosław Paul, Sopot, Poland) composed of 100% micronized apple fiber; white sugar (Polish sugar, Toruń, Poland); mango-passion fruit flavor essence (Browin, Łódź, Poland) with the composition: natural and identical to raw mango and passion fruit flavors, citric acid E330 and mango juice. Probiotic bacteria (Chr. Hansen, Hoersholm, Denmark) were used to produce the inoculum: *Lacticaseibacillus paracassei* L-26, *Lacticaseibacillus casei* 431 and *Lactobacillus acidophilus* LA-5.

### 2.2. Manufacture of Ice Cream Mixes

Sheep’s milk (84.9%), sugar (11%) and flavor essence (0.1%) were mixed and then divided into two parts. Inulin (4%) was added to the first part of the blend and divided into three groups: CP, CC and CA. Inulin (2.5%) and apple fiber (1.5%) were added to the second part and analogously divided into three groups: CPF, CCF and CAF. The milk with additives was mixed and homogenized with a homogenizer (Nuoni GJJ-0.06/40, Zhejiang, China) at 60 °C with a pressure of 20 MPa, and then pasteurized at 85 °C, 1 min. After heat treatment, it was cooled to 37 °C.

The CP and CPF groups were inoculated with a monoculture *Lacticaseibacillus paracasei* L-26, the CC and CCF groups were inoculated with *Lacticaseibacillus casei* 431. The CA and CAF groups were inoculated with *Lactobacillus acidophilus* LA-5. Each starter probiotic culture was previously inoculated in sheep’s milk at 40 °C for five hours. After five hours, 5% (*w*/*w*) of inoculum containing 9 log CFU of g^−1^ bacteria was added to the ice mixes. The fermentation process of the ice cream mixes was carried out in an incubator (Cooled Incubator ILW 115, POL-EKO-Aparatura, Wodzisław Śląski, Poland) at 37 °C for 10 h. After cooling to 5 °C, it was conditioned at the specified temperature for 12 h in an incubator and then froze in a DeLux 48816 freezer (UNOLD AG, Hockeheim, Germany) for 40–50 min with a freezing temperature down to −22 °C. The prepared ice cream was packaged in 100 mL plastic cups and stored at −22 °C for twenty-one days.

The experiment was repeated on three occasions.

### 2.3. Physicochemical Analysis

The chemical composition of the ice cream mixes was determined using a Bentley B-150 Milk and Milk Product Analyzer (Bentley, MN, USA). The determination of the pH value was performed with a FiveEasy digital pH meter (Mettler Toledo, Greifensee, Switzerland) with an electrode InLab^®^Solids Pro-ISM (Mettler Toledo, Switzerland) with an integrated temperature sensor. The content of lactic acid was determined (g of lactic acid L^−1^) by titration of samples of ice cream mixes and dissolved ice cream with 0.1 N NaOH (Chempur, Piekary Śląskie, Poland) according to the method of Jemaa et al. [31]. The melting rate, first dropping time and total melting time was determined at an ambient temperature of 22 °C. Ice cream samples were placed on a wire mesh grid (95 mm diameter, holes 5 × 5 mm, wire thickness 0.5 mm). Then the time until the ice cream wholly dissolved was recorded [32]. Ice cream overrun was estimated as the air volume ratio in frozen ice cream to the importance of melted ice cream expressed in % [33]. Five samples were tested for each ice cream variant, and it was repeated was repeated in triplicate.

### 2.4. Microbiological Analysis

The number of probiotic strains (*Lacticaseibacillus paracasei* L-26, *Lacticaseibacillus casei* 431, *Lactobacillus acidophilus* LA-5) was determined in the ice mix, then immediately after freezing and on the 7th and 21st days of storage at −22 °C. 10 g of each sample was diluted in 90 mL of sterile peptone water solution (0.1%) (BTL Sp. z o.o., Łódź, Poland). Serial dilutions from 1 log CFU g^−1^ to 8 log CFU g^−1^ were made. The inoculation was performed by the plate-deep method using MRS agar (Biocorp, Warszawa, Poland) and incubated anaerobically in a vacuum desiccator at 37 °C for 72 h using the GENbox anaer (Biomerieux, Warszawa, Poland). The cultured probiotic colonies were counted with a colony counter (TYPE J-3, Chemland, Stargard Szczeciński, Poland). The result was expressed as log CFU g^−1^. Five samples were tested for each ice cream variant, and it was repeated was repeated in triplicate.

### 2.5. Color of Ice Cream

The color of the ice cream was measured with a precise colorimeter (model No. 145, Shenzhen, China) using the CIElab system. The following parameters were measured: L*—brightness of ice cream (0—black, 100—white), a* (− a*—shades of green, + a*—shades of red) and b* (− b*—shades of blue, + b*—shades of yellow), C—color saturation and purity and h° as a hue of color. Before analysis, the device was calibrated on a white and black reference standard [34,35]. Five samples were tested for each ice cream variant, and it was repeated was repeated in triplicate.

### 2.6. Organoleptic Analysis

Organoleptic assessment for six groups of ice cream was carried out by a trained team of 15. The samples of ice cream encoded (with a random three-digit code) were analyzed on a nine-point linear scale with marginal, structured markings. The left end of the scale indicated the following features: not very characteristic (appearance); soft (hardness); sandy (smooth); immediate (spreadability); dark (color); hardly perceptible (taste, smell) [36]. The right side of the scale defined the features: very distinctive (appearance), hard (hardness), very smooth (smooth); delayed (spreadability); light (color); very intense (distinguishing features of taste and smell). After analyzing the tested ice cream sample, the panelists were asked to rinse their mouths with water to avoid a cold transfer effect [37].

### 2.7. Statistical Analysis

The mean and standard deviation were calculated using Statistica v. 13.1 (StatSoft, Tulsa, OK, USA). One, two and three-way ANOVA was performed. The significance of differences between the mean values was verified with the Turkey test (*p* < 0.05).

## 3. Results

The chemical composition of sheep milk was: fat 6.56 ± 0.5%, protein 4.60 ± 0.3% and lactose 5.04 ± 0.10%, and was similar to the results obtained by Musial et al. [2]. Olkuska sheep milk was only characterized by a higher lactose content.

### 3.1. Chemical Composition of Ice Cream Mixes

The chemical composition of the ice cream mixes fermented by various strains of probiotic bacteria is presented in Table 1.

The ice cream mixes contained 3.68–3.75% protein, 6.36–6.46% fat and 18.43–18.50% carbohydrates. The ice cream mixes did not differ significantly in terms of protein, fat and carbohydrate content. Partial replacement of inulin with apple fiber and fermentation of ice cream mixes with different probiotic strains do not significantly differentiate their chemical composition. Similar content of protein (3.39%) and carbohydrates (18.39%) in the control milk ice cream was demonstrated by Ismail et al. [38]. Moreover, Balthazar et al. [39] developed and tested sheep’s milk ice cream with a higher fat content (10.03%) and lower protein content (3.2%).

Table 2 shows the moisture results of ice cream after 7 and 21 days of ice cream storage. There were no significant differences in moisture between individual ice cream groups and during storage, which was confirmed by the 3-factor ANOVA (Table 3). These results are in line with the studies by Abdelazez et al. [40] and Ranadheera et al. [41], who showed that the moisture of frozen products does not change significantly during freezer storage.

Table 2 shows the pH values determined before and after the fermentation of the ice mixes. As expected, the 1.5% addition of apple fiber lowered the pH value of ice cream mixes by about 0.2 units before fermentation. The optimal pH for *Lactobacillus* and *Lacticaseibacillus* growth is 5.5–6.0 [42]. The addition of apple fiber favorably reduced the pH value to 6.1, creating conditions similar to the optimal ones for fermentation than in mixtures with inulin only. Reducing the pH value of ice cream with apple fiber might be due to the presence of organic acids. Apple fiber obtained from apple pomace contains organic acids (0.6–0.9%) such as malic, quinic, citric and shikimic acids as well as sugars (7%), fiber (5–6%), trace amounts of protein as well as microelements: calcium, magnesium, iron and potassium [43,44].

As in our study, in the study by Favaro-Trindade et al. [45], the addition of acerola pulp reduced the pH value. In our research, after fermentation, the lower pH value of mixtures with apple fiber (CPF, CCF and CAF) was maintained compared to their counterparts only with inulin (CP, CC and CA). It was shown that using the same fermentation conditions for all mixes, the pH value decreased the most in the fermentation in CP mixtures fermented with *L. paracasei*. Most likely, it results from the specificity of *L. paracasei* because this strain, unlike *L. casei*, ferments lactose to produce L (+) lactic acid, and about 50% of cells also can ferment inulin [46].

Significantly higher pH values were obtained in Lb-fermented CC mixtures. *L. casei* and CA from *Lb. acidophilus* (Table 2). The decrease in the pH value of the mixtures results from the metabolic activity of probiotics in the ice cream mix and the inclusion of prebiotic preparations, i.e., apple fiber. A 3-factor analysis of variance showed that the type of probiotic bacteria significantly affected the pH value and lactic acid content in ice cream (Table 3). In the studies of Soukoulis et al. [47], the pH value of fermented and non-fermented ice cream was between 4.5 and 6.3. In studies by Akca and Akpinar [48], probiotic milk ice cream with the addition of *Lacticaseibacillus rhamnosus* and *Bifidobacterium animalis* ssp. *lactis* Bb-12 was characterized by a pH value of 5.18–5.54 during 90 days of storage. The authors noted the lowest pH value in ice cream with powdered grape seed pulp with prebiotic properties.

In our research, an inverse relationship with the pH value was found for lactic acid content in ice cream mixes and ice cream. In ice cream mixes and ice cream with apple fiber, the lactic acid content was higher by 0.1–0.2 g L^−1^ than in their equivalents with inulin only. These differences persisted during the storage of the ice cream. The lowest content of lactic acid after fermentation was determined in the CA ice cream mix fermented by *Lb. acidophilus.*

The highest amount of lactic acid was found in the mixture of CPF with apple fiber fermented by *L. paracasei*. The analysis of variance shows that lactic acid content is significantly influenced by single factors (bacterial type, storage time or fiber) and interactions between bacterial type and storage time. However, the combined effect of these three factors on the lactic acid content turned out to be insignificant.

Similar results were obtained by Ismail et al. [38] in ice cream enriched with pomegranate peel powder and Abd El-Rashid and Hassan [49], who used Doum palm fruit to make ice cream. According to Farias et al. [50], ice cream’s pH value and acidity do not change during the storage period, regardless of whether they have been fermented or not. As in the studies by Farias et al. [50], our results indicate that extending the storage time from 7 to 21 days does not increase the lactic acid content.

The pH value and acidity of probiotic products can significantly affect the survival of probiotic bacteria cells in fermented ice cream. The addition of growth promoters and prebiotics such as inulin has been shown in many studies to significantly improve the viability of probiotic organisms [51]. Akin et al. [52] studied ice cream containing probiotic bacteria (*Lb. acidophilus* and *Bifidobacterium lactis*), and their results suggest that the addition of inulin stimulates growth *Lb. acidophilus* and *Bifidobacterium lactis*, which improved their survival. Therefore, partial replacement of inulin with apple fiber in our research might also contribute to the differentiation of growth and survival of probiotic bacteria cells.

### 3.2. Microbiological Analysis of Ice Cream

The number of bacterial cells in the mixes and ice cream depending on the type of bacteria, storage time and the addition of apple fiber is presented in Table 4.

After fermentation of the ice cream mixes, bacteria cells ranged from 10.62 log cfu g^−1^ to 12.25 log cfu g^−1^. The addition of apple fiber did not significantly affect the growth of *L. paracasei* in the CPF ice cream mix compared to CP. In turn, this addition contributed to an increase in the number of *Lb. acidophilus* cells by approximately one log cfu g^−1^ in CAF mixtures with apple fiber compared to the CAF mixture with inulin only. It should be mentioned that *Lb. acidophilus,* especially, has a high cytoplasmic buffering capacity (pH 3.72–7.74), which allows it to resist cytoplasmic pH changes and obtain stability under acidic conditions [53,54]. According to Talwalkar and Kailasapathy [55] and Haynes and Playne [56], and Takahashi et al. [57], this stability is influenced by the enzyme H + -ATPase.

In this study, the highest number of bacterial cells was determined in the CC-fermented mixture with *L. casei* with inulin. However, the addition of apple fiber resulted in a reduction in the number of cells by about 1 log CFU g^−1^ in the CCF mixture. These studies indicate that a vital problem is selecting probiotic bacteria for the fermentation of sheep’s milk mixtures because partial replacement of inulin with apple fiber changed the fermentation conditions (e.g., pH value) and differentiated the number of probiotic cells after fermentation.

The resistance of probiotics to freezing damage varies among probiotic strains. Microorganisms that show a better ability to survive under freezing conditions can dehydrate without rupturing the cytoplasmic membranes. Such cells can reduce the number and growth of intracellular ice crystals and thus reduce the heat transfer of their cells; both factors minimize microbial cell damage [58].

In our research, the freezing process reduced the population of probiotic bacteria cells in all ice cream groups. After freezing, significant cell count reductions were observed by 0.8 log cfu g^−1^ in CA ice cream, 1.0 log cfu g^−1^ in CP ice cream and 1.1 log cfu g-1 in CC ice cream with inulin. This means that the survival rate of bacteria when freezing sheep’s milk ice cream mixes depends on the probiotic strain. *Lb. acidophilus* showed the best survival and the lowest reduction of the population after freezing. However, it should be added that this strain multiplied the least when fermenting the CA mix, which resulted in the lowest number of *Lb. acidophilus* cells in CA after fermentation. This is essential information for ice cream producers because fermentation should be extended in industrial production or the starter dose of bacteria increased to obtain a more significant number of *Lb. acidophilus* cells. These treatments are needed to obtain a comparable number of bacterial cells to those obtained in Lb mixtures: *L. paracasei* and *L. casei.*

Freezing mixtures with the addition of apple fiber also resulted in a reduction of viable bacterial cells from 0.8 log CFU g^−1^ in CPF and CAF to 1 log cfu g^−1^ in CCF, compared to the results after fermentation. This means that the reduction in the probiotic population in apple fiber ice cream due to freezing is similar to their inulin counterparts in CCF and CAF ice cream and less only in CPF ice cream. Additionally to the damage caused by probiotic cell freezing, the inclusion of oxygen in the mixture (aeration process) could result in an additional reduction in the number of viable probiotic bacteria cells since the oxygen content, and the redox potential (which is directly proportional to the amount of oxygen) is essential. Most of the probiotic *Lactobacillus* and *Lacticaseibacillus* strains are organisms derived from the intestines with microaerophilic or anaerobic metabolism. Therefore, molecular oxygen and high redox potential values are critical factors for these bacteria [53,59,60].

After seven days of freezing storage at −22 °C, the viability of the probiotics in all ice cream groups decreased, and the population was reduced by another 0.8–1.2 log cfu g^−1^ compared to the number of bacterial cells after freezing. The most intense reduction in the log cycle (1.2 log cfu g^−1^) was found in CC and CCF ice cream with *L. casei* compared to the number of cells determined after freezing. The highest survival after seven days of ice cream storage was characterized by *L. paracasei*, where the reduction in the number of bacterial cells was only 0.8 log cfu g^−1^ in CP ice cream and 0.9 log cfu g^−1^ in CPF ice cream with apple fiber, compared to the number of cells determined after freezing.

In our study, extending the storage time of ice cream from 7 to 21 days did not significantly affect the number of bacterial cells in all ice cream groups. After seven days and 21 days of ice cream storage, the lowest number of bacterial cells was determined in CA and CAF ice cream. After 21 days, the CAF ice cream showed a population reduction of only 0.1 log CFU g^−1^ compared to the 7th day of storage. In contrast, probiotic ice cream stored at −20 °C for 90 days, tested by Turgut and Cakmakci [61], showed a reduction in the number of cells of *Lb. acidophilus* with 0.38 log CFU g^−1^ at this time. Furthermore, in the study by Salem et al. [62], the number of live probiotics in ice cream decreased by 2.23, 1.68, 1.54, 1.23 and 1.77 log CFU g^−1^, respectively, for *Lb. acidophilus*, *B. bifidum*, *Lb. reuteri*, *Lb. gasseri* and *Lb. rhamnosus* within 12 weeks of frozen storage (−26 °C).

The highest number of bacterial cells after 21 days of ice cream storage was determined in CP and CPF ice cream (Table 4). *L. paracasei* was characterized by the best survival rate, which predisposes it to be used to produce probiotic ice cream from sheep’s milk. The conducted 3-factor ANOVA confirms that the survival and the number of probiotic bacteria cells are significantly influenced by all tested factors (type of bacteria, storage time and fiber) and their interactions.

After 21 days of storing ice cream, the number of bacterial cells in each group of ice cream exceeded 8 log cfu g^−1^, which means that the ice cream can be classified as a probiotic food [63,64]. According to Nezhad et al. [65], and at a concentration of 6–7 log cfu g^−1^, the daily therapeutic dose of probiotics is about 8–9 log cfu g^−1^ to compensate for losses during the digestive process. In our research, all ice cream groups meet these recommendations for 21 days of storage.

### 3.3. Physical Analysis of Ice Cream

Detailed analysis of the influence of the bacteria used for fermentation on the aeration of ice cream (Table 5) shows the highest percentage of overrun in the ice cream with *L. paracasei* and *Lb. acidophilus*. *L. casei* ice cream was characterized by significantly lower overrun on the 7th and 21st days of storage. Although the *L. paracasei* ice cream had the greatest overrun, it did not cause a significant reduction in the probiotic population during storage.

The literature reports that grape, peach and apple pomace is valuable source of pectin, especially pectin with many methoxylated groups, which have favorable gelling properties [66]. This type of pectin also improves the aeration ability, which is also confirmed by our research results.

According to Feizi et al. [67], aeration decreases with the increasing addition of chia seed lyophilisate. The authors attribute less aeration of ice cream with a higher chia lyophilisate to the rheological properties of the mixes. Increasing the viscosity of the mixtures reduces the speed of whipping, although a certain level of density is necessary for optimal whipping and air retention. In a similar study, the aeration of ice cream for samples containing basil lyophilisate (0.1 and 0.2% *w*/*w*) was 46.5% and 42.5%, respectively [68].

According to Soukoulis et al. [69], prebiotics affects the incorporation of air and stabilize the foam by increasing the viscosity of the water phase (increasing the concentration of solute and gelling) surrounding the surface between air cells, raising the physical barrier against destabilization of air cells. Numerous ice cream studies have shown that inulin, oligofructose or resistant starch significantly improve air incorporation (aeration) and related properties such as melt resistance and shape retention [23,70]. The melting rate is influenced by many factors, including the chemical composition, the amount of air introduced, the size of the ice crystals, and the structure of the fatty globules formed during freezing [71].

Table 5 shows the first dropping time and total melting rate ice cream tests after 7 and 21 days of storage. It is generally known that the melting rate of ice cream correlates with its aeration [72]. Therefore, the CC ice cream with the lowest overrun had the longest first drop and total melting times. The high melting rate of ice cream with high overrun was probably due to poor air cell stability, air cell size distribution and a network of fat globules formed during freezing. Air, fat globules and ice are the main microstructural components of ice cream and significantly influence the melting or dripping behavior [73,74].

It should be added that the time of the first drop after seven days of storage in the freezer differed significantly depending on the type of bacteria used to ferment the mixture and the addition of apple fiber. The ice cream fermented with *L. casei* had a longer first drop time than that of *L. paracasei* and *Lb. acidophilus*. Extending the storage time from 7 to 21 days significantly reduced the first drop time in all ice cream groups by 57 s in CP, 93 s in CPF, 131 s in CC, 28 s in CCF and 68 s in CA 70 s in CAF. Furthermore, in a study by Bahram-Parvar et al. [71], the melting rate of ice cream increased with increasing shelf life, which may be due to an increase in ice crystal size due to ice recrystallization. Guven et al. [75] found that the addition of various combinations of selected hydrocolloids, such as salep, firewood flour, guar gum and sodium alginate, significantly changes the first drop’s time.

The conducted research shows that the addition of apple fiber significantly shortens the total melting time. It is most likely related to the properties of apple fiber resulting from a lower water retention capacity than inulin. Inulin is a hygroscopic substance; it reacts with water molecules limiting their free movement and stabilizing the ice mixture, reducing meltability [76]. According to Syed et al. [76], the higher content of water-soluble compounds, i.e., inulin and apple fiber, resulted in the enrichment of the liquid phase. Thus, the freezing point was moderately lowered, and the percentage of water in frozen form also decreased, resulting in a lower melting point.

Several researchers have reported conflicting results about the effects of inulin on the melting properties of ice cream. Akbari et al. [26] and Akalin et al. [77] found that low-fat ice cream with different levels of inulin (up to 4%) showed significantly worse melting parameters compared to the control ice cream (no inulin with 10% fat content). In contrast, El-Nagar et al. [78] observed that incorporating inulin into yogurt ice cream mixes resulted in a reduction of the melting time. Akalin and Erisir [79] also indicated that using both inulin and oligofructose in ice cream improves the melting properties of ice cream with a 4% fat content. Akin et al. [52] confirmed that adding inulin delays the melting of ice cream because inulin can act as a stabilizer by binding water molecules.

However, the presence of pectin in apple fiber may also contribute to the cryopreservation of ice cream by controlling the mobility of water molecules in the non-frozen aqueous phase due to its thermodynamic incompatibility with the current separation phase proteins [25]. Goh et al. [80] report that protein–protein and protein–polysaccharide at the interface with fat globule/air cell and dispersed components in the serum phase can also influence melting properties [67].

In our study, the difference in total melting time was most significant in the *L. casei* ice cream, where the melting time was 1206 s shorter in the CCF ice cream with apple fiber compared to the CCF ice cream with inulin. On the other hand, CP and CPF ice cream fermented with *L. paracasei* had a significantly lower total melting time. In these ice cream, the addition of apple fiber reduced the total melting time by 488 s compared to CP ice cream with inulin only. Extending the shelf life from 7 to 21 days significantly reduced the total melting time only in *L. casei*-fermented ice cream (CC and CCF) and *Lb. acidophilus* (CA and CAF). However, in ice cream with *L. paracasei*, the storage time did not significantly affect the total melting time.

### 3.4. Color Parameters of Ice Cream

The results describing the color parameters of ice cream and ice cream mixes are presented in Table 6.

The brightest was the CP mixes fermented with *L. paracasei*, while the slightly darker CC mixes fermented with *L. casei* and CA with *Lb. acidophilus*. Partial replacement of inulin with 1.5% of apple fiber resulted in a significant darkening of the color of ice cream mixes. Depending on the type of bacterial mixtures used for fermentation, the addition of apple fiber increased the value of the L* parameter from 13.2 to 16.6.

After seven days of freezing storage in CPF and CAF ice cream, the L* brightness of the ice cream increased compared to the blends. The remaining ice cream (CP, CC, CA and CCF) also tended to increase the brightness of the ice cream, but the differences were not significant. This color change in ice cream samples was due to ice crystals and air bubbles inside the ice cream formed during whipping during freezing [81].

According to Nozière et al. [82], inulin molecules interact with casein micelles, which together with fat globules are responsible for light scattering and, consequently, for a high L* value. Extending the storage time to 21 days also increased the L* parameter in all ice cream groups. Higher L* values indicate a brighter, more white color of the ice cream.

The a* (+ a—redness, − a—green) parameter in CP, CC and CA ice cream mixes and ice cream with inulin only took negative values, while CPF, CCF and CAF mixes and ice cream with apple fiber-positive values. This means that in ice cream mixes and ice cream with inulin only (CP, CC and CA), a greater proportion of green color was observed, while in mixes and ice cream with apple fiber (CPF, CCF and CAF), the red color was dominant. After the 7th day of storage, the proportion of green color in ice cream with inulin (CP, CC and CA) increased compared to the a* parameter determined in ice cream mixes (CP, CC and CA). Moreover, in the ice cream with apple fiber, an intensification of the red color was observed compared to the a* value determined in the mixes (CPF, CCF and CAF).

Extending the storage time of ice cream with apple fiber from 7 to 21 days increased the proportion of red color. The analyzed ice cream mixes and ice cream with inulin and apple fiber were characterized by a high ratio of yellow (+ b*). The minor balance of yellow was found in the CA ice cream mix fermented by *Lb. acidophilus*. On the other hand, the most yellow CP mixes and ice cream fermented by *L. paracasei*. This effect is confirmed by the studies of Acevedo-Martinez et al. [83], which inform that the type of probiotic bacteria and the 5% addition of fructo-oligosaccharides may significantly impact the color of the analyzed products.

A 1.5% addition of apple fiber resulted in the intensification of the yellow color in CPF, CCF and CAF mixtures. Moreover, after 7 and 21 days of freezing storage, the yellow color was found. These results are confirmed by the analysis of variance, which shows that the type of bacteria, storage time and addition of apple fiber and the interactions of these factors significantly influenced the intensity of yellow color. Furthermore, when analyzing the results of the C color saturation and the h° hue of ice cream mixes and ice cream, a significant effect of apple fiber was found.

Apple fiber is characterized by a beige color, which is formed during the drying of apple pomace. During drying, the enzyme polyphenol oxidase causes oxidation of polyphenols resulting in browning of the powder. The same effect is obtained from the non-enzymatic browning reaction, i.e., the reaction of sugars with amino acids, occurring at high speed at an elevated temperature and with a water content of about 30% [84,85]. The color components of dried apple fiber, determined by Rząca and Witrowa-Rajchert [30], indicate a large proportion of red and yellow, which explains the role of these colors in forming the color of ice cream in our research.

### 3.5. Organoleptic Analysis

The results of the organoleptic evaluation of ice cream on days 7 and 21 of freezer storage are presented in Table 7.

The most characteristic appearance of milk ice cream was that of ice cream with CA and CP inulin, both after 7 and 21 days of freezing storage. This is probably because the use of apple fiber caused a significant reduction in L* brightness, and the fiber ice cream was darker and redder than inulin only ice cream. The ANOVA, also, confirms that the addition of fiber had a significant effect on the appearance of sheep’s milk ice cream.

In the Akalin et al. [77] study, ice cream with apple fiber also scored lower for appearance due to its darker color. Crizel et al. [86], also, observed that the orange peel fiber used lowered the acceptance of ice cream among consumers.

Ice cream hardness depends on the aeration and size of the ice crystals. The hardness is the greater the larger the ice crystals, while with the increase in overrun, the hardness decreases [87,88]. Moreover, in our research, the highest hardness was found in the least aerated CC and CCF ice cream. On the other hand, CPF and CAF ice cream, with the highest percentage of overrun, turned out to be the softest.

According to Franck [89], the added inulin of ice cream improves its texture due to its ability to bind water molecules and form a gel network of molecules. In our research, ice cream only with inulin (CP, CC and CA) was also characterized by higher hardness than their counterparts with the addition of apple fiber. Additionally, El-Nagar et al. [78] found in yogurt ice cream that the addition of inulin increased the hardness of the ice cream. Moreover, in the studies of Tiwari et al. [90], the milk ice cream with 4% inulin was harder than the control ice cream. Akalin and Erisir [79] and Di Criscio et al. [22] also demonstrated the effect of inulin addition on ice cream hardness. In ice cream tested by Hashemi et al. [91], an increase in hardness was also observed in ice cream where hydrogenated vegetable oil was replaced with inulin.

The addition of apple fiber in our study reduced the hardness of ice cream compared to ice cream with inulin only, but these differences were not significant. According to Bahram-Parvar et al. [71], hardness is a parameter that indicates the smoothness and wateriness of ice cream samples [92]. In our research, the smoothness of ice cream was significantly influenced by the addition of apple fiber, which reduced the feeling of smoothness and creaminess during consumption, increasing their sandiness at both times. The significant effect of the addition of apple fiber is confirmed by the ANOVA (Table 3).

According to Abdullah et al. [93] and Soukoulis and Tzia [94], the most important parameter determining the acceptance of frozen desserts is their taste. In our research, the sweetest ice cream turned out to be CA ice cream from *Lb. acidophilus* on both dates. This is most likely due to the low lactic acid content of 0.50–0.51 g L^−1^ in CA ice cream compared to other ice cream groups. Moreover, the panelists, analyzing the taste of sheep’s milk ice cream (Table 7), found that ice cream with 4% inulin was characterized by a sweeter taste than ice cream with apple fiber. The least sweet were the CAF, CPF and CCF ice cream with 1.5% apple fiber at both storage times. This is probably related to the acids in apple fiber and the low pH and high lactic acid content of the ice cream. The panelists indicated that the addition of apple fiber positively increased the taste and aroma of the mango-passion fruit, but the differences in the notes were not significant.

As in the studies by Turgut and Cakmaci [61] also in our studies, fermentation guided by *L. paracasei, L. casei*, *Lb. acidophilus*, the addition of inulin and apple fiber did not introduce any foreign taste and smell to the ice cream. Furthermore, according to Salem et al. [62], no foreign taste was found in the milk ice cream with probiotic strains during storage. The conducted ANOVA (Table 3) shows that the storage time and type of bacteria used for fermentation and their interactions do not significantly affect the organoleptic characteristics of probiotic ice cream from sheep’s milk. Only the addition of fiber significantly influenced the appearance and smoothness of the ice cream.

## 4. Conclusions

Symbiotic ice cream with acceptable physicochemical and organoleptic characteristics may be produced from sheep milk of the Olkuska breed. Moreover, partial replacement of inulin with apple fiber contributed to increased overrun in the ice cream and to a shorter run-off time of the first drop and the total melting time. The addition of apple fiber stimulated the growth of *L. paracasei* and *Lb. acidophilus*. The best survival rate was found for *L. paracasei*. Results demonstrated the effects of using apple fiber and various types of bacteria in milk ice cream, mainly probiotics that have not yet been studied and used in sheep’s milk ice cream. Partial replacement of inulin with apple fiber is, also, a way to reduce production costs.

## Figures and Tables

**Table 1:** Chemical composition of ice cream mixes samples after fermentation.

Chemical Composition	CP	CPF	CC	CCF	CA	CAF
Protein, %	3.69 ^a^ ± 0.08	3.71 ^a^ ± 0.05	3.68 ^a^ ± 0.03	3.72 ^a^ ± 0.01	3.70 ^a^ ± 0.02	3.75 ^a^ ± 0.08
Fat, %	6.36 ^a^ ± 0.21	6.41 ^a^ ± 0.04	6.38 ^a^ ± 0.02	6.46 ^a^ ± 0.10	6.42 ^a^ ± 0.10	6.38 ^a^ ± 0.02
Carbohydrates, %	18.45 ^a^ ± 0.06	18.48 ^a^ ± 0.07	18.43 ^a^ ± 0.02	18.45 ^a^ ± 0.02	18.48 ^a^ ± 0.11	18.50 ^a^ ± 0.11

Mean ± standard deviation; n (for each trial) = 5; ^a^—mean marked with the same letter do not differ at *p* ≤ 0.05. CP: sample with 4% inulin and *L. paracasei* L-26, CPF: sample with 2.5% inulin, 1.5% apple fiber and *L. paracasei* L-26, CC: sample with 4% inulin and *L. casei* 431, CCF: sample with 2.5% inulin, 1.5% apple fiber and *L. casei* 431, CA: sample with 4% inulin and *Lb. acidophilus* LA-5 and CAF: sample with 2.5% inulin, 1.5% apple fiber and *Lb. acidophilus* LA-5.

**Table 2:** Lactic acid content, pH value and moisture of ice cream mixes and ice creams during storage.

Properties	Storage Time (Days)	CP	CPF	CC	CCF	CA	CAF
Moisture, %	7	71.79 ^Aa^ ± 0.19	71.98 ^Aa^ ± 0.23	71.53 ^Aa^ ± 0.44	71.55 ^Aa^ ± 0.13	71.61 ^Aa^ ± 0.23	71.65 ^Aa^ ± 0.20
	21	71.73 ^Aa^ ± 0.11	71.82 ^Aa^ ± 0.04	71.51 ^Aa^ ± 0.43	71.63 ^Aa^ ± 0.06	71.68 ^Aa^ ± 0.17	71.74^Aa^ ± 0.36
pH	0	6.47 ^Cd^ ± 0.01	6.18 ^Cb^ ± 0.01	6.38 ^Cc^ ± 0.01	6.13 ^Da^ ± 0.01	6.37 ^Bc^ ± 0.01	6.14 ^Ca^ ± 0.01
	1	4.42 ^Ac^ ± 0.00	4.19 ^Aa^ ± 0.01	4.55 ^Ad^ ± 0.01	4.35 ^Ab^ ± 0.00	5.29 ^Af^ ± 0.01	4.83 ^Ae^ ± 0.02
	7	4.49 ^Ba^ ± 0.03	4.34 ^Ba^ ± 0.06	4.58 ^ABb^ ± 0.04	4.51 ^Cb^ ± 0.06	5.31 ^Ad^ ± 0.01	4.88 ^ABc^ ± 0.06
	21	4.51 ^Bc^ ± 0.01	4.31 ^Ba^ ± 0.01	4.59 ^Bd^ ± 0.01	4.44 ^Bb^ ± 0.00	5.29 ^Af^ ± 0.01	4.88 ^Be^ ± 0.01
Lactic acid, g L^−1^	1	0.77 ^Ad^ ± 0.01	0.97 ^Bf^ ± 0.00	0.63 ^Ab^ ± 0.01	0.85 ^Be^ ± 0.01	0.48 ^Aa^ ± 0.01	0.70 ^Ac^ ± 0.00
	7	0.79 ^Ad^ ± 0.00	0.92 ^Af^ ± 0.10	0.74 ^Bc^ ± 0.01	0.83 ^Ae^ ± 0.00	0.50 ^Aa^ ± 0.01	0.69 ^Ab^ ± 0.02
	21	0.78 ^Ac^ ± 0.04	0.89 ^Ae^ ± 0.12	0.74 ^Bc^ ± 0.01	0.82 ^Ad^ ± 0.01	0.51 ^Aa^ ± 0.02	0.69 ^Ab^ ± 0.02

Mean ± standard deviation; n (for each trial storage time) = 5; ^a,b,c,d,e,f^—mean values denoted in rows by different letters differ statistically significantly at *p* ≤ 0.05; ^A,B,C,D^—mean values in columns obtained for a given parameter denoted by different letters differ significantly (*p* ≤ 0.05). CP: sample with 4% inulin and *L. paracasei* L-26, CPF: sample with 2.5% inulin, 1.5% apple fiber and *L. paracasei* L-26, CC: sample with 4% inulin and *L. casei* 431, CCF: sample with 2.5% inulin, 1.5% apple fiber and *L. casei* 431, CA: sample with 4% inulin and *Lb. acidophilus* LA-5 and CAF: sample with 2.5% inulin, 1.5% apple fiber and *Lb. acidophilus* LA-5. Storage time: 0—ice cream mixes before fermentation, 1—ice cream mixes after fermentation, 7—ice cream after 7 days and 21—ice cream after 21 days.

**Table 3:** Analysis of variance (ANOVA) *p*-values on the effects of storage time and type of bacteria and fiber on color, pH, lactic acid, overrun, bacteria appearance, hardness, smoothness, sweet taste, additives taste, off taste, odor additives and off odor of ice cream.

Properties	Type of Bacteria *p*-Values	Storage Time (Days) *p*-Values	Fiber *p*-Values	Type of Bacteria × Storage Time *p*-Values	Type of Bacteria × Fiber *p*-Values	Storage Time × Fiber *p*-Values	Type of Bacteria × Storage Time × Fiber *p*-Values
L*	↑0.0003	↑0.0013	↑0.0000	↑0.0002	↑0.0023	↑0.0409	↑0.0429
a*	↑0.0042	↑0.0000	↑0.0000	↑0.0011	↑0.0000	↑0.0000	↑0.0087
b*	↑0.0000	↑0.0000	↑0.0000	↑0.0000	↑0.0000	↑0.0229	↑0.0095
C*	↑0.0000	↑0.0000	↑0.0000	↑0.0000	↑0.0000	↑0.0050	↑0.0126
h*	↑0.0000	↑0.0445	↑0.0000	↑0.0192	↑0.0000	↑0.0210	↑0.0013
pH	↑0.0000	↑0.0092	↑0.0000	n.s. 0.5101	↑0.0000	n.s. 0.1481	n.s. 0.1938
Lactic acid	↑0.0000	↑0.0197	↑0.0000	n.s. 0.1708	↑0.0000	n.s. 0.3194	n.s. 0.7370
Moisture	n.s. 0.1445	n.s 0.9054	n.s. 0.1250	n.s. 0.7038	n.s. 0.5281	n.s. 0.1147	n.s. 0.8499
Overrun	↑0.0000	↑0.0400	↑0.0000	↑0.0201	↑0.0000	↑0.0300	↑0.0050
First drop	↑0.0000	↑0.0000	↑0.0000	↑0.0000	↑0.0000	↑0.0000	↑0.0000
Complete melting times	↑0.0012	↑0.0421	↑0.0000	↑0.0357	↑0.0000	↑0.0190	↑0.0038
Bacteria	↑0.0000	↑0.0467	↑0.0020	↑0.0148	↑0.0018	↑0.0075	↑0.0477
Appearance	n.s. 0.2095	n.s. 0.8393	↑0.0161	n.s. 0.4821	n.s. 0.8393	n.s. 0.3936	n.s. 0.7393
Hardness	n.s. 0.7721	n.s. 0.7320	n.s. 0.0752	n.s. 0.6358	n.s. 0.4151	n.s. 0.4705	n.s. 0.9648
Smoothness	n.s. 0.6102	n.s. 0.0538	↑0.0000	n.s. 0.8722	n.s. 0.5308	n.s. 0.2277	n.s. 0.8627
Sweet taste	n.s. 0.1617	n.s. 0.4032	n.s. 0.1286	n.s. 0.7476	n.s. 0.7831	n.s. 0.8888	n.s. 0.9072
Additives taste	n.s. 0.8766	n.s. 0.0532	n.s. 0.7218	n.s. 0.9038	n.s. 0.8437	n.s. 0.9432	n.s. 0.9537
Off taste	n.s. 0.1329	n.s. 0.7729	n.s. 0.1532	n.s. 0.9193	n.s. 0.1329	n.s. 0.7729	n.s. 0.9193
Odor additives	n.s. 0. 8592	n.s. 0.4555	n.s 0.5052	n.s. 0.9013	n.s. 0.9305	n.s. 0.1462	n.s. 0.9305
Off odor	n.s. 0.5317	n.s. 0.4276	n.s. 0.4276	n.s. 0.5327	n.s. 0.5317	n.s. 0.4276	n.s. 0.5317

Storage time (days) = interaction↑; Type of bacteria × fiber = interaction↑; Storage time × fiber = interaction ↑; Type of bacteria × storage time × fiber = interaction↑; indicates significant effect *p* < 0.05; n.s.—no significant effect ↑.

**Table 4:** Viable counts of probiotic bacteria in ice creams and ice creams mixture (log cfu g^−1^).

Storage Time	CP	CPF	CC	CCF	CA	CAF
1	11.99 ^Cc^ ± 0.80	12.08 ^Cc^ ± 0.80	12.25 ^Cd^ ± 0.10	12.07 ^Cc^ ± 0.72	10.64 ^Ca^ ± 0.69	11.62 ^Cb^ ± 0.79
2	10.99 ^Bbc^ ± 0.85	11.19 ^Bd^ ± 0.88	11.17 ^Bd^ ± 0.14	11.05 ^Bc^ ± 0.77	9.80 ^Ba^ ± 0.75	10.74 ^Bb^ ± 0.83
7	10.18 ^Ac^ ± 0.85	10.20 ^Ac^ ± 0.87	9.97 ^Ab^ ± 0.81	9.85 ^Ab^ ± 0.81	8.86 ^Aa^ ± 0.72	9.74 ^Ab^ ± 0.61
21	10.18 ^Ac^ ± 0.80	10.17 ^Ac^ ± 0.86	9.88 ^Ab^ ± 0.76	9.85 ^Ab^ ± 0.77	8.83 ^Aa^ ± 0.72	9.68 ^Ab^ ± 0.76

Mean ± standard deviation; n (for each trial storage time) = 5; ^a,b,c,d^—mean values denoted in rows by different letters differ statistically significantly at (*p* ≤ 0.05); ^A,B,C^—mean values in columns obtained for a given parameter denoted by different letters differ significantly (*p* ≤ 0.05). CP: sample with 4% inulin and *L. paracasei* L-26, CPF: sample with 2.5% inulin, 1.5% apple fiber and *L. paracasei* L-26, CC: sample with 4% inulin and *L. casei* 431, CCF: sample with 2.5% inulin, 1.5% apple fiber and *L. casei 431*, CA: sample with 4% inulin and *Lb. acidophilus* LA-5 and CAF: sample with 2.5% inulin, 1.5% apple fiber and *Lb. acidophilus* LA-5. Storage time: 1—after fermentation, 2—directly after freezing, 7—after 7 days and 21—after 21 days.

**Table 5:** Overrun, first dropping time, and total melting rate of ice cream in 7 and 21 days of storage.

Properties	Storage Time (Days)	CP	CPF	CC	CCF	CA	CAF
Overrun, %	7	50.55 ^Ac^ ± 0.92	69.90 ^Ae^ ± 0.20	23.35 ^Aa^ ± 0.92	32.55 ^Ab^ ± 1.91	49.10 ^Ac^ ± 0.28	65.76 ^Ad^ ± 0.20
	21	52.67 ^Ac^ ± 0.47	69.46 ^Ad^ ± 0.65	22.70 ^Aa^ ± 0.42	35.79 ^Bb^ ± 0.76	52.34 ^Bc^ ± 1.00	69.95 ^Bd^ ± 0.06
First drop, s	7	957 ^Bc^ ± 7.78	719 ^Bb^ ± 8.38	1580 ^Bd^ ± 4.04	977 ^Bc^ ± 8.82	954 ^Bc^ ± 8.49	647 ^Ba^ ± 7.07
	21	900 ^Ad^ ± 8.79	626 ^Ab^ ± 5.36	1449 ^Af^ ± 4.75	949 ^Ae^ ± 9.90	886 ^Ac^ ± 9.19	577 ^Aa^ ± 7.78
Complete melting times, s	7	5566 ^Ab^ ± 48.08	5078 ^Aa^ ± 60.10	7381 ^Bd^ ± 58.69	6175 ^Bc^ ± 49.50	6280 ^Bc^ ± 57.28	5565 ^Ab^ ± 79.20
	21	5543 ^Ac^ ± 35.16	5073 ^Aa^ ± 22.33	6978 ^Af^ ± 31.11	6014 ^Ad^ ± 15.86	6171 ^Ae^ ± 20.31	5477 ^Ab^ ± 29.50

Mean ± standard deviation; n (for each trial storage time) = 5; ^a,b,c,d,e,f^—mean values denoted in rows by different letters differ statistically significantly at (*p* ≤ 0.05); ^A,B^—mean values in columns obtained for a given parameter denoted by different letters differ significantly (*p* ≤ 0.05). CP: sample with 4% inulin and *L. paracasei* L-26, CPF: sample with 2.5% inulin, 1.5% apple fiber and *L. paracasei* L-26, CC: sample with 4% inulin and *L. casei* 431, CCF: sample with 2.5% inulin, 1.5% apple fiber and *L. casei* 431, CA: sample with 4% inulin and *Lb. acidophilus* LA-5 and CAF: sample with 2.5% inulin, 1.5% apple fiber and *Lb. acidophilus* LA-5. Storage time: 7—ice cream after 7 days and 21—ice cream after 21 days.

**Table 6:** Color parameters of ice cream sample in the ice cream mixture during storage.

Color	Storage Time (Days)	CP	CPF	CC	CCF	CA	CAF
L*	1	83.36 ^Ae^ ± 0.65	66.72 ^Aa^ ± 0.86	81.97 ^Ad^ ± 0.94	68.38 ^Ab^ ± 0.35	82.43 ^Ade^ ± 0.62	69.22 ^Ac^ ± 0.56
	7	83.67 ^Ad^ ± 0.09	71.48 ^Bb^ ± 0.25	82.64 ^Ac^ ± 0.07	69.81 ^Aa^ ± 0.62	82.62 ^Ac^ ± 0.03	71.40 ^Bb^ ± 0.83
	21	84.86 ^Ac^ ± 0.85	77.20 ^Ca^ ± 0.15	83.31 ^Ac^ ± 0.79	69.83 ^Ab^ ± 1.84	87.37 ^Bd^ ± 0.10	76.43 ^Ca^ ± 1.17
a*	1	−1.88 ^Aa^ ± 0.12	3.57 ^Ad^ ± 0.18	−1.06 ^Bc^ ± 0.05	4.40 ^Ae^ ± 0.21	−1.23 ^Ab^ ± 0.06	3.41 ^Ad^ ± 0.21
	7	−2.09 ^Bb^ ± 0.04	3.94 ^Bc^ ± 0.09	−1.28 ^Ab^ ± 0.01	4.54 ^Ad^ ± 0.15	−1.25 ^Ab^ ± 0.02	3.88 ^Ac^ ± 0.33
	21	−2.08 ^Ba^ ± 0.02	4.74 ^Cc^ ± 0.05	−1.29 ^Ab^ ± 0.05	5.32 ^Bd^ ± 0.08	−1.41 ^Ab^ ± 0.20	4.92 ^Bc^ ± 0.13
b*	1	16.49 ^Ac^ ± 0.44	19.43 ^Ad^ ± 0.43	14.98 ^Ab^ ± 0.42	20.18 ^Ae^ ± 0.57	14.40 ^Aa^ ± 0.08	21.87 ^Af^ ± 0.28
	7	19.72 ^Bb^ ± 0.26	20.11 ^Bc^ ± 0.21	15.06 ^Ba^ ± 0.04	20.73 ^Ac^ ± 0.33	15.02 ^Ba^ ± 0.05	22.07 ^Ad^ ± 0.42
	21	20.69 ^Bb^ ± 0.60	22.01 ^Cc^ ± 0.09	15.79 ^Ca^ ± 0.54	22.82 ^Bc^ ± 0.30	15.06 ^Ba^ ± 0.41	22.95 ^Bc^ ± 0.33
C*	1	16.63 ^Ab^ ± 0.44	19.83 ^Ac^ ± 0.42	14.04 ^Aa^ ± 0.41	20.07 ^Ac^ ± 0.59	14.45 ^Aa^ ± 0.08	21.43 ^Ad^ ± 0.21
	7	19.87 ^Bc^ ± 0.06	20.61 ^Bd^ ± 0.06	16.12 ^Bb^ ± 0.04	20.14 ^Acd^ ± 0.44	14.97 ^Ba^ ± 0.04	21.37 ^Be^ ± 0.07
	21	20.82 ^Cc^ ± 0.59	23.63 ^Ce^ ± 0.10	16.85 ^Cb^ ± 0.54	22.60 ^Bd^ ± 0.31	15.13 ^Ba^ ± 0.41	22.61 ^Bd^ ± 0.35
h*	1	91.61 ^Ae^ ± 0.62	75.07 ^Ab^ ± 0.57	92.09 ^Ae^ ± 0.50	74.52 ^Ba^ ± 0.51	89.90 ^Ad^ ± 0.24	76.18 ^Ac^ ± 0.61
	7	90.45 ^Ad^ ± 0.53	74.39 ^Aa^ ± 0.54	92.06 ^Ae^ ± 0.25	74.32 ^Aba^ ± 0.97	89.68 ^Ac^ ± 0.17	76.89 ^Ab^ ± 0.72
	21	90.55 ^Ae^ ± 0.30	74.26 ^Ab^ ± 0.12	92.36 ^Af^ ± 0.13	73.39 ^Aa^ ± 0.09	89.37 ^Ad^ ± 0.71	76.67 ^Ac^ ± 0.22

Mean ± standard deviation; n (for each trial storage time) = 5; ^a,b,c,d,e,f^—mean values denoted in rows by different letters differ statistically significantly at (*p* ≤ 0.05); ^A,B,C^—mean values in columns obtained for a given parameter denoted by different letters differ significantly (*p* ≤ 0.05). CP: sample with 4% inulin and *L. paracasei* L-26, CPF: sample with 2.5% inulin, 1.5% apple fiber and *L. paracasei* L-26, CC: sample with 4% inulin and *L. casei* 431, CCF: sample with 2.5% inulin, 1.5% apple fiber and *L. casei* 431, CA: sample with 4% inulin and *Lb. acidophilus* LA-5 and CAF: sample with 2.5% inulin, 1.5% apple fiber and *Lb. acidophilus* LA-5. Storage time: 1—after fermentation, 7—after 7 days and 21—after 21 days.

**Table 7:** Organoleptic characteristics of ice cream on 7 and 21 days of storage.

Properties	Storage Time (Days)	CP	CPF	CC	CCF	CA	CAF
Appearance	7	7.67 ^Abc^ ± 0.63	6.00 ^Aa^ ± 1.28	7.50 ^Bb^ ± 0.52	5.50 ^Aa^ ± 0.76	8.00 ^Ac^ ± 0.89	5.50 ^Aa^ ± 0.76
	21	7.25 ^Ac^ ± 0.56	5.50 ^Aab^ ± 0.73	6.00 ^Ab^ ± 0.56	5.50 ^Aab^ ± 0.91	7.00 ^Ac^ ± 0.41	4.75 ^Aa^ ± 0.71
Hardness	7	5.83 ^Aab^ ± 0.98	4.50 ^Aa^ ± 1.76	8.17 ^Aa^ ± 0.17	7.17 ^Aa^ ± 1.14	5.83 ^Ab^ ± 0.75	4.67 ^Aa^ ± 1.16
	21	5.25 ^Aab^ ± 0.50	4.00 ^Aa^ ± 0.16	8.75 ^Aa^ ± 0.22	7.00 ^Aa^ ± 0.16	5.25 ^Aa^ ± 0.71	4.75 ^Ab^ ± 0.89
Smoothness	7	7.17 ^Ac^ ± 0.75	4.00 ^Ab^ ± 0.67	6.50 ^Ac^ ± 0.38	3.83 ^Ab^ ± 0.23	7.00 ^Ac^ ± 0.89	3.83 ^Aa^ ± 0.17
	21	7.25 ^Ab^ ± 1.71	5.00 ^Aa^ ± 0.82	7.25 ^Ab^ ± 1.71	4.00 ^Aa^ ± 0.82	7.25 ^Ab^ ± 1.71	4.50 ^Aa^ ± 1.29
Sweet taste	7	5.33 ^Aa^ ± 0.51	4.83 ^Aa^ ± 0.47	5.33 ^Aa^ ± 0.51	5.17 ^Aa^ ± 0.83	6.17 ^Ab^ ± 0.33	5.17 ^Aa^ ± 0.32
	21	4.75 ^Aa^ ± 0.26	4.50 ^Aa^ ± 0.29	5.00 ^Aa^ ± 0.82	4.50 ^Aa^ ± 0.96	6.15 ^Ab^ ± 0.56	5.25 ^Aa^ ± 0.26
Additives taste	7	4.50 ^Aa^ ± 0.55	5.00 ^Aa^ ± 0.90	4.50 ^Aa^ ± 0.55	4.67 ^Aa^ ± 0.47	4.67 ^Aa^ ± 0.21	4.33 ^Aa^ ± 0.27
	21	5.25 ^Aa^ ± 0.96	5.50 ^Aa^ ± 1.00	5.50 ^Aa^ ± 0.29	5.75 ^Aa^ ± 0.96	5.25 ^Aa^ ± 0.96	5.25 ^Aa^ ± 1.71
Off taste	7	1.00 ^Aa^ ± 0.00	1.00 ^Aa^ ± 0.00	1.00 ^Aa^ ± 0.00	1.00 ^Aa^ ± 0.00	1.00 ^Aa^ ± 0.00	1.17 ^Aa^ ± 0.41
	21	1.00 ^Aa^ ± 0.00	1.00 ^Aa^ ± 0.00	1.00 ^Aa^ ± 0.00	1.00 ^Aa^ ± 0.00	1.00 ^Aa^ ± 0.00	1.25 ^Aa^ ± 0.50
Odor additives	7	2.50 ^Aa^ ± 0.14	3.00 ^Aa^ ± 0.15	2.33 ^Aa^ ± 0.15	3.33 ^Aa^ ± 0.11	2.33 ^Aa^ ± 0.21	2.83 ^Aa^ ± 0.17
	21	3.25 ^Aa^ ± 0.50	3.00 ^Aa^ ± 0.82	3.00 ^Aa^ ± 0.00	2.75 ^Aa^ ± 0.50	3.00 ^Aa^ ± 0.00	2.75 ^Aa^ ± 0.50
Off odor	7	1.00 ^Aa^ ± 0.00	1.00 ^Aa^ ± 0.00	1.00 ^Aa^ ± 0.00	1.00 ^Aa^ ± 0.00	1.00 ^Aa^ ± 0.00	1.00 ^Aa^ ± 0.00
	21	1.00 ^Aa^ ± 0.00	1.00 ^Aa^ ± 0.00	1.00 ^Aa^ ± 0.00	1.00 ^Aa^ ± 0.00	1.00 ^Ba^ ± 0.00	1.00 ^Aa^ ± 0.00

Mean ± standard deviation; n (for each trial storage time) = 15; ^a,b,c^—mean values denoted in rows by different letters differ statistically significantly at (*p* ≤ 0.05); ^A,B^—mean values in columns obtained for a given parameter denoted by different letters differ significantly (*p* ≤ 0.05). CP: sample with 4% inulin and *L. paracasei* L-26, CPF: sample with 2.5% inulin, 1.5% apple fiber and *L. paracasei* L-26, CC: sample with 4% inulin and *L. casei* 431, CCF: sample with 2.5% inulin, 1.5% apple fiber and *L. casei* 431, CA: sample with 4% inulin and *Lb. acidophilus* LA-5 and CAF: sample with 2.5% inulin, 1.5% apple fiber and *Lb. acidophilus* LA-5. Storage time: 7—ice cream after 7 days and 21—ice cream after 21 days.

## Data Availability

Not applicable.
